# RNA Extraction from Animal and Human's Cancerous Tissues: Does Tissue Matter?

**Published:** 2015

**Authors:** Ali Akbar Samadani, Novin Nikbakhsh, Sadegh Fattahi, Roghayeh Pourbagher, Seyyed Mohsen Aghajanpour Mir, Narges Mousavi Kani, Zeinab Abedian, Haleh Akhavan-Niaki

**Affiliations:** 1 *Cellular and Molecular Biology Research Center, Babol University of Medical Sciences, Babol, Iran.*; 2 *Department of Surgery, Babol University of Medical Sciences, Babol, Iran.*

**Keywords:** RNA extraction, frozen tissue, cancerous tissues

## Abstract

The reliability of gene expression profiling, based technologies and methods to find transcriptional differences representative of the original samples is influenced by the quality of the extracted RNA. Hence, RNA extraction is the first step to investigate the gene expression and its function. Consequently, the quality of extracted RNA is really significant. Correspondingly, this research was accomplished to optimize the RNA extraction methods and compare the amounts of tissue or quality of tissue. Relatively, the cancerous tissue of human stomach in fresh and frozen conditions and also the mouse fresh tissue were studied. Some factors like the amount of samples, efficacy differences of diverse extraction buffers (TriPure, Trizol) and also the efficacy of b-mercaptoethanol were compared and investigated. The results indicated that the less amount (1-2 mg) compared to other amounts (2-5 mg, 5-15 mg) yielded the best quality and the RNA bands (5S, 18S, 28S) were observed perfectly. Relatively, comparing and measuring some kinds of buffers (Trizol, TriPure) indicated no difference in RNA extraction quality. The last investigated factor was the effect of b- mercaptoethanol which was used along with TriPure to remove the RNAse. Conclusively, no effective impression was observed.

The development of protocols for RNA extraction from tissue samples facilitates gene expression studies on all kinds of samples (paraffin- embedded, fresh and frozen) with known clinical and practical conclusion (1). Correspondingly, for many years, human tissue samples acquired upon surgery have been routinely fixed in formalin and embedded in paraffin for long- term storage or frozen and in some cases have been used freshly (2). Consequently, most research and/ or medical centers have important tissue archives allowing molecular study and long-term follow up of many kinds of neoplasms or even unique tumors. One major restriction of formalin-fixed paraffin-embedded samples for gene expression profiling is the high instability of RNA which can easily be degraded prior to formalin fixation, making RNA extraction from such samples a problematic challenge (3). Moreover, formalin fixation creates cross-linking between nucleic acids and proteins and adds mono-methyl to amino groups of all RNA bases (N-CH_2_OH); leading consequently to methylene bridging between neighboring bases (N-CH_2_-N), creating therefore another barrier to further transcriptomics investigation (4).

Immediate freezing of fresh tissue samples preserves good quality RNA for gene expression studies. However, this procedure is not routinely performed in most hospitals, therefore limiting the number of valuable large frozen tissue biobanks worldwide. Conspicuously, frozen tissue bank containing tumor samples may be biased in their collection as such tumors must be sufficiently large and palpable in order to allow tissue excision and freezing for the bank collection (5). Correspon-dingly, there are many documented procedures for nucleic acids extraction among which using kits is becoming the most universal one. In this account, there are currently multiple commercially available kits. In most of them, the RNA is extracted by spin column purification according to similar basic principles: deparaffinization (if previously paraffin- embedded) followed by cell disruption with proteinase K, which is capable of efficiently degrading proteins that were covalently cross- linked with each other and RNA, thereby allowing more efficient RNA extraction than achieved by the use of chaotropic agents such as guanidinium salt (6). After proteinase K incubation, RNA is isolated by alcohol precipitation and use chaotropic salt such as guanidinium thiocyanate or guanidinium chloride in a spin column purification step (7).

Remarkably, RNA extraction methods from tissue samples are the key components of downstream molecular profiling of tumors, especially in cancer biobanking for further transcriptome analysis either for research or diagnosis. A major limitation of current routinely used procedures is that many of them are not developed and/ or validated for the RNA extraction from tissue samples. Contrary to blood or cell culture samples, tissue samples are often heterogeneous and may vary in composition. Indeed some tissues such as breast may have a high fat content and low cell number, while others like muscle might be very fibrous or may have high cell density like liver samples. It is therefore not recommended to use a universal extraction procedure for all tissue types (8). The quality of RNA extracted from tissues may also be variable and depends on many factors, including time of removal from the patient to freezing or fixation, tissue thickness and storage conditions (9). When possible, it is recommended to use fresh frozen tissue for extracting RNA of high integrity (3, 10).

The aim of this study was to optimize RNA extraction method and compare the tissue quality and quantity.

## Materials and methods

**Tissues**

Twenty samples of human gastric tumors and 20 samples of mouse muscle and liver in different amounts (1 to 15 mg) were chosen for RNA extraction. All human specimens were collected in frozen and fresh conditions and transferred by nitrogen tank from surgery room to the laboratory directly. Relatively, DEPC (Diethylpyruvate carbonate) was used for removing the RNase for all surgery sets.

**RNA extraction**

Firstly, liquid nitrogen was added and the samples were ground separately. Then, either 1 ml Trizol reagent (Invitrogen, USA) or 1 ml TriPure (Roche, USA) was added. Upon completion of the harvest procedure, the homogenates were transferred to empty RNase free falcon tubes stored on ice. To prevent microbial contamination and subsequent RNase contamination, disposable gloves were always used and good sterile technique and methods were practiced when handling samples. Also, the gloves were changed frequently. Then, ethanol (250 μl), isopropanol (500μl), chloroform (200 μl) were added respectively. Subsequently, once b–mercaptoethanol was added to the homogenate to remove the RNase. Ultimately, the quality of RNA was checked on agarose gel electro-phoresis to observe the 5S, 18S and 28S bands.

## Results

**Effect of tissue amount**

**Fig. 1:**
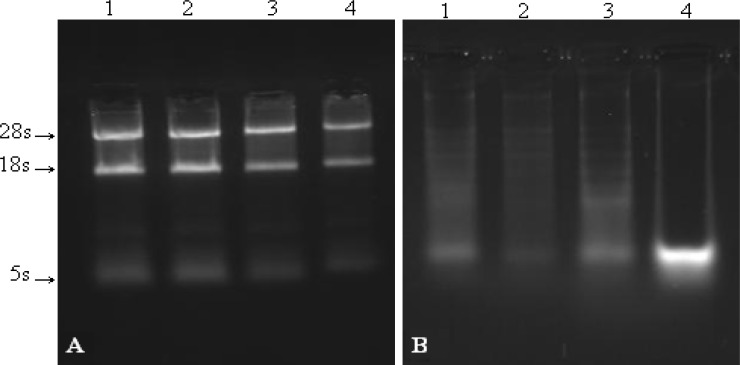
The effect of increasing the amount of tissues. A: lanes 1 and 2: 1-2 mg, gastric cancer; lane 3: 2-5 mg, liver tissue; lane 4: 2-5 mg, muscle tissue. B: lanes 1 and 2: 5-15 mg, gastric cancer; lane 3: 5-15 mg, liver tissue; lane 4: 5-15 mg, muscle tissue

For the first step, we compared and investigated the effect of tissue amount. In this account, we chose the different amounts of diverse tissues such as gastric tumors of human, liver and muscle of mouse varying between 1-5 mg (Figure 1A) and 5-15 mg (Figure 1B).

As shown in Figure 1B, by increasing the amount of tissue, the quality of the RNA bands decreased. Remarkably, 28S and 18S are degraded.

**Effect of Trizol and TriPure**

**Fig. 2:**
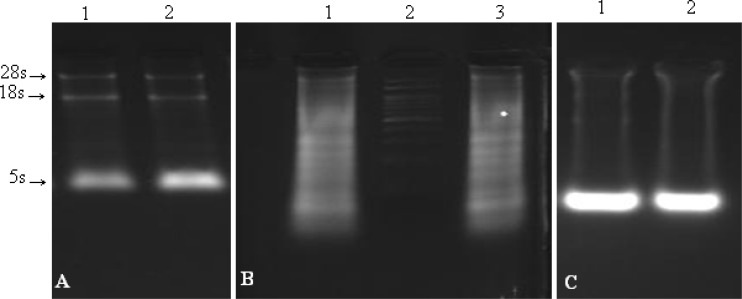
Comparison of Trizol and TriPure effect. A: Lanes 1 (liver) and 2 (muscle) treated with TriPure and Trizol, respectively (the approximate amount of tissues: 2-5 mg). B: lanes 1 and 2 (gastric tumors) treated with Trizol and lane 3 (gastric tumors) treated with TriPure (the approximate amount of tissues: 5-10 mg). C: lanes 1 (liver) and 2 (muscle) treated with Trizol and TriPure, respectively (the approximate amounts of tissue: 10-15 mg).

For the second investigation, the effect of some RNA buffers like: Trizol and TriPure, on all kind of mentioned tissues were compared. Conclusively, no impressive difference was observed (Figure 2).

**Fig. 3:**
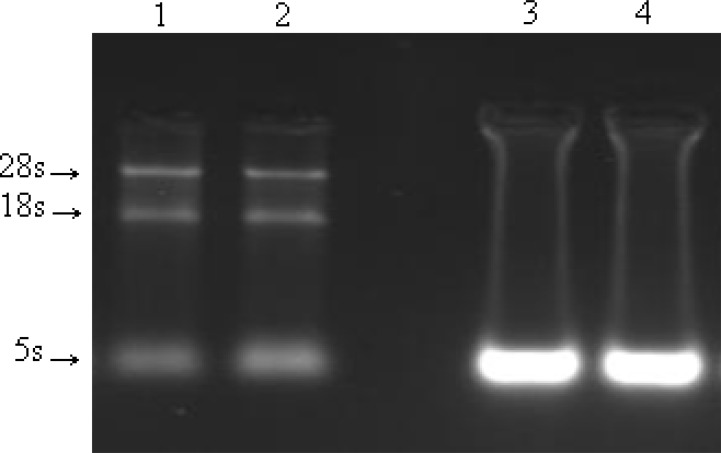
Effect of b-mercaptoethanol. Lanes 1 and 2 (gastric tumors fresh and frozen respectively) (the approximate amount of tissues: 2-5 mg), lane 3 (liver tissue) and lane 4 (muscle tissue). B-mercaptoethanol was added in lanes 1 and 3 (the approximate amount of tissues: 10-15 mg).

**Effect of B-mercaptoethanol**

B-mercaptoethanol was added in all kinds of samples in order to break the disulfide bands and remove the RNAse effect. Evidently, there was no significant effect of b-mercaptoethanol on RNA extraction (Figure 3).

## Discussion

RNA's expression's level is a remarkable factor of cell and tissue physiological condition and the measuring suitability of gene expression is severely related to the quality and quantity of RNA (11, 12). The success in every RNA analysis is dependent on the RNA purity and also quality and quantity of extracted RNA (13).

The challenges of undertaking studies comprising human full-thickness skin tissues are different. In addition to dealing with numerous regulatory research centers, the researcher must obtain samples from consenting patients and overcome the challenges of working with small, restricted biopsies. Traditionally, the mechanical disruption of skin samples has been severe, needing large amounts of beginning tissue that is ground using mortar and pestle (14) or alternatively by a tissue homogenizer. Although the current trend of homogenization is an improvement for softer tissues, full -thick tissue is naturally resistant to shearing forces resulting in incomplete sample disruption and sample loss (15). The aim of this study was to look for conditions which may influence maximum efficiency when extracting our molecules of interest. It is essential to do this reliably with all biopsy samples, so that persistently repeatable conclusions are acquired. The process by which these molecules are extracted must be compatible and leading to usable data in downstream techniques (4). Indeed, in expression studies, the variability between individual patient samples can cause a high signal background, which masks the less abundantly expressed genes that may be unregulated. This is of particular concern in systems that utilize complex tissue samples containing multiple cell types and cell-to-cell contact. Approximately, due to advanced molecular techniques, it is possible to perform extensive transcriptomic studies with microgram quantities of RNA. Thus, care must be taken to preserve the integrity of the sample during preparation steps. We used an optimized extraction method for small amounts of tissue samples and found that the amount of initial tissues is critical for preserving RNA integrity. Relatively, b-mercaptoethanol has an important and remarkable role in RNA extraction from animal and human tissues. Additionally, b-mercaptoethanol is used to contribute to demolish RNases that may be present and will degrade the RNA. B– mercaptoethanol is a decreasing agent that will reduce the disulfide bonds of the RNases, in consequence devastating the conformation and the functionality of the enzyme (10). The absence of any improvement in RNA quality in the presense of b- mercaptoethanol in our experiments suggests that the extraction buffer used (e.g. Trizol and TriPure) contain either this component or a similar chemical which inhibits RNases. As coldness can preserve the nucleic acids from degradation, storing the samples in liquid nitrogen or freezing and also working in a cold room is a main factor and necessary. Taking into account the amounts of samples are very effective and important. As we demonstrated, the lesser amount between 1- 2 mg had a better consequence than the other ranges. This is because of the consequent lesser amount of RNase in the samples. For Trizol and Tripure, no remarkable modifications were observed. It means that in the conditions used, these buffers have an equal effect in RNA extraction. Muscle and liver tissues of mouse and also gastric tumors both in frozen and fresh conditions were investigated separately. Remarkably, the best quality was affiliated to muscle tissues. 

In conclusion, the effect of coldness (working in a cool room or in ice buckets) and the use of the least amount (the least volume) yielded the best quality and all protocols were able to extract RNA with a minimally acceptable quality from all kinds of tissues (gastric tumors, liver and muscle tissues of mouse). Also, there was no impressive influence of b-mercaptoethanol and no difference between Trizol and TriPure. To some extent, if a selected protocol fails to extract RNA from a tissue in the first step, then another extraction and so an alternative protocol should be employed before excluding this from further investigation.
